# The Effects of the Addition of a New Airway Clearance Device to Chest Physiotherapy in Children with Cystic Fibrosis Pulmonary Exacerbations

**DOI:** 10.34763/jmotherandchild.20202403.2013.d-20-00008

**Published:** 2021-01-26

**Authors:** Katarzyna Walicka-Serzysko, Magdalena Postek, Natalia Jeneralska, Aleksandra Cichocka, Justyna Milczewska, Dorota Sands

**Affiliations:** 1 Cystic Fibrosis Department, Institute of Mother and Child, Warsaw, Poland; 2 Cystic Fibrosis Centre, Pediatric Hospital, Dziekanow Lesny, Poland

**Keywords:** cystic fibrosis, pulmonary exacerbation, chest physiotherapy, airway clearance device, pulmonary function test, lung clearance index

## Abstract

**Background:**

Chest physiotherapy plays a crucial role in managing cystic fibrosis, especially during pulmonary exacerbations. This study evaluated the effects of adding a new airway clearance device to chest physiotherapy in subjects with cystic fibrosis hospitalised due to pulmonary exacerbations.

**Methods:**

This prospective open-label study was carried out at the Pediatric Cystic Fibrosis Centre in Poland between October 2017 and August 2018. Cystic fibrosis patients aged 10 to 18 years who were admitted to the hospital and required intravenous antibiotic therapy due to pulmonary exacerbations were consecutively allocated (1:1) to either chest physiotherapy alone or chest physiotherapy with a new airway clearance device (Simeox; PhysioAssist). Patients performed spirometry and multiple-breath nitrogen washout for lung clearance index assessment upon admission and prior to discharge.

**Results:**

Forty-eight cystic fibrosis patients were included (24 in each group). Spirometry parameters in both groups improved significantly after intravenous antibiotic therapy. A significant improvement in the maximum expiratory flow at 25% of forced vital capacity was observed only in the group with a new airway clearance device (p < 0.01 vs. baseline). Trends towards a lower lung clearance index ratio were similar in both groups. No adverse events were observed in either group.

**Conclusions:**

Spirometry parameters increased significantly in cystic fibrosis patients treated for pulmonary exacerbations with intravenous antibiotic therapy and intensive chest physiotherapy. The new airway clearance device was safe and well tolerated when added to chest physiotherapy and may be another option for the treatment of pulmonary exacerbation in cystic fibrosis.

## Introduction

Cystic fibrosis (CF) is a multisystem disease. However, in most cases, quality of life and longevity are determined by the progression of lung disease. Alternating periods of stability and pulmonary exacerbation (PEx) contribute to gradual clinical deterioration and the worsening of lung function. Respiratory failure is still the most frequent cause of death in CF patients, which is why slowing the progression of lung disease is central to CF management strategies [1]. Furthermore, chronic inflammation and infection with pathogens such as *Staphylococcus aureus, Pseudomonas aeruginosa* and *Aspergillus fumigatu*s require daily treatment. PEx are serious events for CF patients. They are associated with major clinical consequences such as the irreversible and progressive loss of lung function [2, 3, 4], increased risk for future exacerbations [5], reduced health-related quality of life and increased risk of death [6]. To prevent lung function decline, PEx must be treated properly. Management should include an intensification of chronic daily therapies [7] (chronic medications, airway clearance techniques [ACTs]) and antibiotics [8].

Effective mucus clearance is essential to reduce symptoms and optimise treatment in CF, particularly during PEx. There

are a number of ACTs that have been utilised in patients with CF, including an active cycle of breathing techniques, autogenic drainage, positive expiratory pressure (PEP), high pressure PEP, oscillating PEP, postural drainage and percussion and physical exercise. Guidelines state that chest physiotherapy (CP) and ACTs should be individually tailored to the need and preference of every patient and that no approach has shown superiority over another [9]. New and modified approaches to physiotherapy are always being investigated in order to increase the effectiveness, tolerability and safety of treatment. Assessment of the benefits of these new approaches is based on improvements in clinical parameters and lung function.

A new airway clearance device (ACD) that uses a pneumatic vibratory stimulus has been developed (Simeox; PhysioAssist, France). The action of this device is based on the rheological and thixotropic properties of mucus. It spreads a vibratory pneumatic signal in the bronchial tree during relaxed exhalation by disseminating a succession of very short negative air pressure pulses of adjustable constant volume at a frequency of 6 or 12 Hz. The device is designed to modify mucus viscosity and elasticity, mobilising mucus in the distal airways and transporting it to more proximal airways for productive expectoration.

This study evaluated the effects of this new ACD on lung function in subjects with CF who were hospitalised due to PEx. Secondary objectives were to examine safety, tolerability, quality of life and patient satisfaction with the device.

## Methods

### Study design

This prospective open-label trial was carried out at the Cystic Fibrosis Centre in Poland from October 2017 to August 2018. The study was conducted according to the principles outlined in the Declaration of Helsinki and Good Clinical Practice. The study protocol was approved by the local ethics committee and all patients (or their legally appointed and authorised representative) provided written informed consent before the enrolment in the study.

### Patients

Patients aged 10 to 18 years with CF who were admitted to the hospital and required IV antibiotic therapy due to PEx were eligible for the study and invited to participate. The diagnosis of CF was based on current criteria [1, 10, 11], and PEx was defined according to Fuchs criteria [12]. Additional inclusion criteria included the ability to perform lung function tests (multiple-breath nitrogen washout [N_2_MBW] and spirometry). Key exclusion criteria included contraindications to bronchial CP, hemoptysis, pneumothorax, recent chest injury or surgery, history of transplantation, heart disease and history of any other illness or any clinical condition that, in the opinion of the investigator, might confound the cooperation or the results of the study or pose an additional risk to the subject in using study technology.

### Interventions

Consecutive patients were allocated (1:1 ratio) to conventional CP alone (CP group) or CP plus the ACD (Simeox; PhysioAssist) (CP + device group). Over a two-week hospitalization period, CP or CP + device sessions were performed three times a day, with device parameters individually adjusted for each patient by a physiotherapist. Morning treatment sessions with a physiotherapist included administration of bronchodilators, nebulization of hypertonic saline (both groups), and autogenic drainage for 20 min (CP group) or autogenic drainage for 20 min with an ACD session (CP + device group). Afternoon sessions with a physiotherapist consisted of physical activity, nebulised administration of dornase alfa (Pulmozyme) (both groups), and autogenic drainage for 20 min (CP group) or autogenic drainage for 20 min with an ACD session (CP + device group). Evening treatment sessions in both groups included bronchodilator administration, nebulization of hypertonic saline, and oscillating positive expiratory pressure (PEP) therapy (Aerobica, Flutter, Acapella) using an individualised number of repetitions (drainage time of 20 min). ACD settings were determined individually for each patient (3–5 series of 6–10 exhalations per session).

The ACD consists of a turbine to generate negative air pressure, a vibration generator and a microcontroller to control vibration frequency and all user interfaces. The device is connected to a breathing system that including a mouthpiece, a single-patient breathing chain filter with a flexible tube and a machine protection filter. Control of the device is via a touchscreen application that allows the frequency and delivered power to be varied.

All patients also received IV antibiotics and any other chronic medical treatment.

### Assessments

Clinical data, including genotype, the presence pancreatic insufficiency and the presence of *P. aeruginosa* infection, were obtained from hospital records. Vital signs and clinical assessment (including PEx according to Fuchs criteria) were evaluated during hospitalization. Patients performed spirometry and N_2_MBW upon admission and prior to discharge. At the same time, they also completed the Cystic Fibrosis Questionnaire-Revised (CFQ-R). Sputum or throat swab samples were collected and cultured for various bacterial species, including *S. aureus* and *P. aeruginosa*, and fungal species including *A. fumigatus*. At the end of treatment patients were asked to complete treatment satisfaction questionnaires (comfort, pain, fatigue, ease of use). N_2_MBW was performed to allow calculation of the lung clearance index (LCI), which is a lung function measure that can detect damage in small and large airways prior to changes in lung function determined by spirometry [13].

Spirometry (Jaeger Vyntus IOS; CareFusion, Hochberg, Germany) was performed according to American Thoracic Society/European Respiratory Society (ATS/ERS) criteria [14, 15]. Reference equations from the Global Lung Function Initiative (GLI) were used to calculate *z*-scores and percent predicted values for forced expiratory volume in 1 sec (FEV_1_), forced vital capacity (FVC) and maximum expiratory flow at 25% of FVC (MEF_25_).

N_2_MBW tests were performed with the Exhalyzer-D (EcoMedics AG, Duernten, Switzerland, software version 3.2.0**)**. An MBW test was considered successful if there were at least two or more technically acceptable tests in accordance with guidelines in the ERS/ATS consensus statement [16]. All LCI results were expressed as the mean of at least two technically acceptable results obtained during one session; usually the session included three or more tests.

Safety was determined based on the occurrence of adverse events, serious adverse events and data from the patient follow-up questionnaire.

### Statistical analysis

Data were analysed using STATISTICA version 13.1. Descriptive statistics are used to present data (median with interquartile range, mean ± standard deviation [SD], or number of patients [percentage]). Qualitative data were compared using Fisher test or Chi-2 test. Quantitative data were compared using the unpaired or paired Student t-test, Wilcoxon test or non-parametric test (Mann-Whitney or Welch signed rank test) according to normality of distribution and group comparison. A *p*-value of <0.05 was considered to indicate statistical significance.

## Results

### Study population

Over the one-year recruitment period, 48 patients with CF met the inclusion criteria and were enrolled in the study (24 each in the CP and CP + device groups). There were a number of statistically significant differences between the two treatment groups at baseline (**Table 1**). A full dataset was obtained from all patients in the CP group, but six patients in the CP + device group were not able to perform N_2_MBW due to extended wash-in and wash-out periods; these subjects did not differ from the rest of study population.

**Table 1:** Characteristics of the study population.

Characteristic	CP (n=24)	CP + device (n=24)	p-value
Age, years	13 (12–16)	14 (13–17)	NS
Male, n (%)	16 (66.7)	7 (29.2)	<0.01
Height, cm	161.0±12.3	159.3±14.7	NS
BMI, kg/m^2^	18.9±2.5	18.2±5.9	NS
BMI z-score	–0.2±0.8	–0.9±0.9	<0.05
LCI	12.3±4.0	14.4±3.8^*^	<0.05
LCI z-score	(7.312.9 –19.5)	(10.915.0 –50.0)^*^	NS
FEV_1_, % predicted	76.0±20.6	66.7±19.2	<0.05
FEV_1_, L	2.4±0.9	1.9±0.8	<0.05
FVC, % predicted	87.8±18.8	77.7±18.6	<0.05
FVC, L	3.2±1.1	3.0±1.2	<0.05
FEV_1_/FVC, %	86.8±10.2	77.0±14.1	<0.01
FEV_1_/FVC z-score	-1.58±1.07	-2.42±1.14	<0.02
MEF25-75, % predicted	58.7±28.2	38.0±24.9	<0.01 NS
*Pseudomonas aeruginosa*, n (%)	8 (33)	13 (54)	
F508del homozygous, n (%)	11 (46)	14 (58)	NS
Pancreatic insufficiency, n (%)	23 (96)	23 (96)	NS

Values are median (interquartile range), mean ± standard deviation, or number of patients (%).

BMI, body mass index; CP, chest physiotherapy; FEV, forced expiratory _1_volume in 1 second; FVC, forced vital capacity; LCI, lung clearance index; MEF 25-75, maximum expiratory flow at 25-75% of FVC; NS, not statistically significant.

* n=18 in the CP + device group.

### Lung function

**Figure 1:**
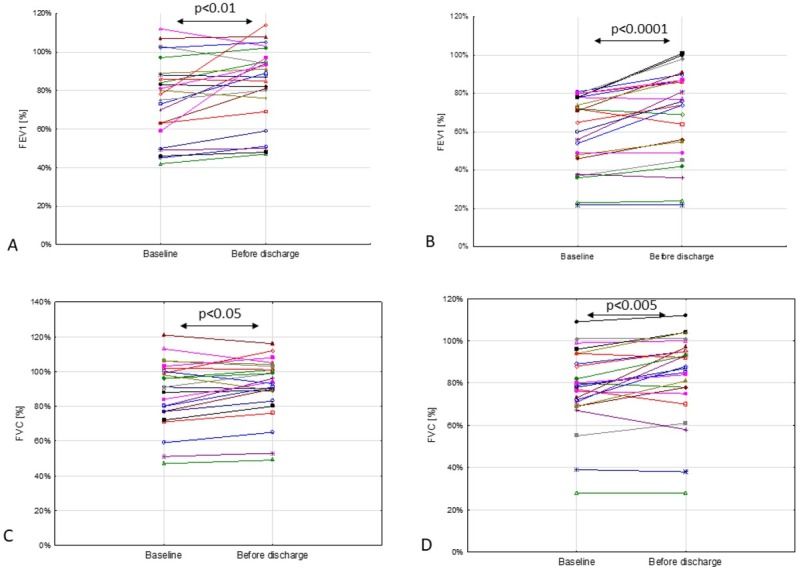
Changes in forced expiratory volume in 1 second (FEV_1_) with treatment in the CP (**A**) and CP + device (**B**) groups (individual patient data). Changes in forced vital capacity (FVC) with treatment in the CP (**C**) and CP + device (**D**) groups (individual patient data).

**Figure 2:**
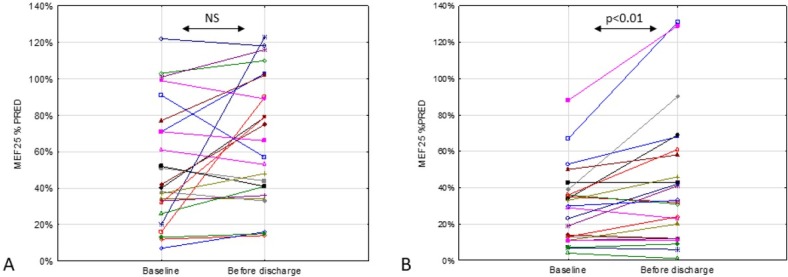
Change in maximal expiratory flow at 25% of forced vital capacity (MEF_25_) with treatment in the CP (**A**) and CP + device (**B**) groups (individual patient data).

In both groups, FEV_1_ and FVC (Fig. 1) increased after IV antibiotic therapy. Changes from baseline were statistically significant in both treatment groups (*p* < 0.01 for FEV_1_ and *p* < 0.05 for FVC in the CP group; *p* < 0.0001 for FEV_1_ and *p* < 0.005 for FVC in the CP + device group) (**Table 2**). MEF at 50% and 75% of FVC (MEF_50_ and MEF_75_) also increased significantly from baseline in both treatment groups (*p* < 0.005 and *p* < 0.05 in the CP group; *p* < 0.005 and *p* < 0.01 in the CP + device group). In contrast, MEF_25_ values only significantly increased from baseline in the CP + device group (*p* < 0.01) (Fig. 2). There was a trend of LCI *z*-score improvement in both groups, but this was not statistically significant (**Table 2**).

**Table 2:** Pulmonary function tests.

	CP				CP + device			
	Baseline	Before discharge	Mean change from baseline to discharge	p-value	Baseline/	Before discharge	Mean change from baseline to discharge	p-value
LCI:								
Mean ± SD	12.3±4.0	11.5±3.8	–0.8±2.0	0.030	14.4±3.8^*^	13.7±4.1^*^	–0.7±2.4^*^	NS
% change			–5.0±16.3				-4.4±13.5^*^	
LCI z-score								
Mean ± SD	19.2±22.1	18.1±24.6	-1.1±7.9	NS	29.0±27.5^*^	23.9±27.5^*^	–5.1±14.8^*^	NS
% change			–5.3±49.5				–10.1±21.8^*^	
FEV_1_, % predicted:								
Mean ± SD	76.0±20.6	83.3±19.9	7.3±12.0	0.007	66.7±19.2	69.6±23.5	9.0±9.1	<0.001
% change			11.5±17.5				14.3±14.2	
FVC, % predicted								
Mean ± SD	87.8±18.8	91.1±16.9	3.3±6.9	0.030	77.7±18.6	83.2±20.4	5.5±8.1	0.003
% change			4.4±7.7				7.1±11.2	
FEV_1_/FVC, %								
Mean ± SD	86.8±10.2	91.6±8.2	4.8±9.0	0.003	77.0±14.1	82.2±16.5	5.0±6.0	<0.001
% change			6.6±14.1				7.7±7.8	
FEV_1_/FVC z-score								
Mean ± SD	-1.58±1.07	-1.06±0.97	0.52±0.95	0.007	-2.42±1.142	-1.93±1.28	0.49±0.64	0.001
% change			-18.0±68.3				- 47.4±120.4	
MEF_25_, %	52.0±32.9	65.9±34.7	13.9±29.6	NS	29.4±20.7	41.8± 35.5	12.4±18.8	0.006
MEF_50_, %	73.5±34.9	87.6±35.5	14.1±29.5	0.022	48.3±32.2	59.0±33.1	10.7±18.0	0.008
MEF_75_, %	75.7±25.1	90.7±29.1	15.0±23.1	0.004	66.0±27.9	78.3±31.2	12.3±18.2	0.003
MEF_25-75_, %	58.7±28.2	73.0±28.8	14.3±27.1	0.006	38.0±24.9	48.0±31.2	10.0±14.1	<0.002

Values are mean ± standard deviation.

CP, chest physiotherapy; FEV, forced expiratory volume in 1 second; FVC, forced vital capacity; LCI, lung clearance index; MEF_125, 50, 75, 25-75_, maximum expiratory flow at 25, 50, 75% or 25-75% of FVC, respectively; NS, not statistically significant; SD standard deviation.

* n=18 in the CP + device group.

### Symptoms and quality of life

During the study, all patients showed a gradual improvement in their general condition and in the resolution of PEx symptoms. Quality of life improved during hospitalization, with no difference in overall and domain scores (e.g. cough, sputum production or difficulty breathing) between treatment groups.

### Side effects

No side effects were observed for either group. Use of the ACD was not rated as painful by any patients; all children claimed that it was very easy or easy to relax during exhalation and breath was more fluent. Most subjects (79%) did not feel fatigue during or after treatment.

### Ease of use and patient preference

The ACD was rated as very easy or easy to use by 91% of patients, and 83% of them quickly learned the new technique. All subjects felt comfortable during drainage, and tolerance of treatment was good or very good. After training, 91% of patients thought that they could use the ACD on their own, and 87% stated that they would like to use it at home. Overall, 62% of patients preferred the ACD over CP, and all said that they would recommend the device to other patients.

## Discussion

The results of this study showed significant improvement in spirometry parameters in CF patients treated for PEx with IV antibiotic therapy and intensive CP, with or without a new ACD. Small airway function appeared to improve to a greater extent when the device was added to CP. Treatment was safe and well tolerated, and patients were satisfied with device therapy. Based on our findings, the mechanism benefit of the airway clearance device in CF is presumed to be the more efficient mobilization of mucus in the distal airways as well as the proximal bronchi.

We used pulmonary function tests (spirometry and N_2_MBW) to evaluate the effects of the interventions in this study. FEV_1_ is considered to be the cornerstone of pulmonary function testing. It is the most widely used method for clinical monitoring of lung function in children, adolescents and adults with CF. FEV_1_ shows strong correlation with airway wall thickness and mucus plugging, both features of larger airway obstruction [17]. However, this parameter has some limitations. Spirometry is very difficult to perform for young children, and FEV_1_ is not sensitive to the small airway damage that occurs during early disease progression [17, 18]. Therefore, in early CF, there can be considerable disturbance of a large numbers of small airways, with relatively little effect on FEV_1_ [17].

In our study, spirometry parameters reflecting larger airways (i.e. FEV_1_, FVC, MEF_75_ and MEF_50_) increased significantly after IV antibiotic therapy in both groups of patients who were hospitalised due to PEx of CF. These improvements were seen in both treatment groups. Interestingly, MEF_25_ (a surrogate endpoint for assessing obstruction of peripheral bronchi and bronchioles) only improved from the baseline in the CP + device group (*p* < 0.01). This suggests that use of the ACD may reduce peripheral bronchial obstruction, thus improving function in small as well as large airways. MEF_25_ has also been shown to improve significantly during drug treatment of CF [19], despite the high intra- and inter-individual variability of this parameter.

MEF_25_, indicating the maximum exhalatory flow after 75% of the air has been exhaled from the lung, is a parameter that reflects changes in the peripheral airways [20]. Deterioration in MEF_25_ over time has been shown to occur as symptoms increase, and this parameter has been shown to correlate well with trapped air on computed tomographic scanning [21]. Van der Giessen and colleagues showed that improvement in MEF_25_ with dornase alfa was significantly greater when treatment was given before versus after ACT, while there were no changes in other spirometric parameters [22]. In another study, deterioration in MEF_25-75_ in children and adolescents with CF was detected before any changes were seen in FEV_1_ and FVC [23]. In a study using data from 15,700 spirometric test cycles in CF patients and healthy children, MEF_25_ was shown to be a more sensitive indicator of disease progression than FEV_1_ or FVC, and there was no increase in MEF_25_ with age [24]. However, a significant amount of variability in MEF_25_ has been reported [22]. Therefore, this variability could impact the interpretation of test results and influence conclusions about changes during therapy.

In contrast to FEV_1_, LCI derived from MBW is a measurement of lung function that is capable of detecting early airway disease in CF [13]. This test measures the tidal breaths needed to remove an inert tracer gas present in the lungs. LCI is sensitive to damage in both large and small airways before a noticeable drop in spirometry measurements [13], enabling detection of early airway disease in CF [25]. The LCI provides an indication of ventilation heterogeneity based on how many breaths it takes to wash a tracer gas out of the lungs during tidal breathing [13]. LCI values increase in parallel with increasing lung disease severity [13], and LCI correlates with the risk of PEx in patients with CF [26]. In addition, LCI has been shown to decrease significantly based on pulmonary symptoms and improve with antibiotic treatment [27]. LCI also appears to worsen during cough episodes and pulmonary exacerbations in children with CF, but not in healthy children [28].In our study, the LCI showed a downward trend over time and reductions from baseline were clinically relevant, but LCI *z*-score decreases did not reach statistical significance in either group. This may have been a result of the small sample size especially in the device group, with 6/24 patients (mean FEV_1_ 53% of predicted; range 23%-59%) unable to perform the test required to determine LCI. Indeed, LCI is not practical for patients with advanced lung disease (FEV_1_ < 60% of predicted) due to profound ventilation heterogeneity and extended wash-in and wash-out periods [17], which was the issue with patients unable to complete the test in our study. It is also possible that some patients have “peeled off” areas that have remained “stuck” and that this increased the ventilation heterogeneity. In CF chronic lung disease, treatment of PEx with IV antibiotics and intensive CP can lead to the recruitment of additional lung units not involved in LCI measurement previously, causing ventilation inhomogeneity [29]. The heterogeneous response of LCI to antibiotic therapy during PEx has been reported previously, with reductions of 2.2–5.5%, similar in magnitude to the changes seen in our study [27, 29, 30, 31, 32, 33]. It was emphasised that LCI alone shouldn’t be used to assess the short-term response to IV antibiotic therapy. While some patients in our study experienced a large reduction in LCI, this parameter remained unchanged or worsened in many others. In summary, LCI response to therapy for PEx is heterogeneous in CF patients. The overall improvement is small, and results are often discordant with FEV_1_.

As noted above, LCI and FEV_1_ measure different aspect of lung physiology. FEV_1_ mainly reflects large airways function and will be affected by changes in airway tone, mucus accumulation in the airways and air trapping. Previous studies with computed tomography demonstrated improvement of all these factors after treatment of PEx, but the main factor contributing to that improvement may vary between patients [30, 34]. Other studies suggested that mucus plugging – and especially large-airway mucus plugging – changes more than other aspects of CF lung disease during antibiotic therapy [35]. In contrast, LCI is a more sensitive marker for peripheral airway abnormalities and their heterogeneities. However, LCI results do not correlate to changes in bronchomotor tone, which is the case for FEV_1_, as demonstrated by studies investigating LCI before and after administration of bronchodilators [36, 37]. LCI response could also have been affected by previously closed compartments contributing to the measurement.

The influence of physiotherapy in CF on LCI measurements remains unclear. In one small study, the authors concluded that there was no relationship between the timing of physiotherapy sessions and LCI values [38], and another suggested that LCI can change markedly when assessed after physiotherapy [39]. Although there are a number of factors in our study that make it dificult to interpret the LCI results, we feel that it was important to include this measure of small airway function in our analyses.

Questionnaire responses in our study suggest that patients considered the new ACD to be efective in removing pulmonary tract secretions, well tolerated, safe and convenient to use. Patients reported that the efort associated with respiratory drainage was slighter when using the ACD compared with conventional physiotherapy techniques; most of the children did not feel any pain or fatigue. The device was judged useful in the hospital and was able to be used autonomously during daily respiratory rehabilitation. All patients gave a positive response to the question whether they thought they could use the device at home. Manual CP is often time-consuming and burdensome for patients and can be quite tiring. Therefore, the right level of patient acceptability is an important feature of the new device that would facilitate its incorporation into clinical practice as part of routine patient care.

This study provides useful initial data on the use of a new ACD in hospitalised CF patients with PEx. However, the study has a number of limitations. The study had a non-randomised design where treatment allocation was done on a consecutive, alternate 1:1 basis. This lack of randomisation to treatment likely contributed to the imbalance in baseline characteristics between groups. The open-label nature of the study means that sources of bias cannot be excluded. In addition, the sample size was small, and the duration of follow-up was short. The two weeks of the follow-up period provided good information about the efects of the ACD in hospitalised patients, but longer-term efects in the ambulatory setting need to be studied. In addition, a variety of factors can afect the outcome of PEx in CF, and these may have confounded our results. The value and reliability of spirometry to assess treatment efects in CF has been questioned [40], but this remains a commonly used tool, and its inclusion facilitates comparison of our results with other studies. Finally, use of antibiotic therapy during the PEx likely influenced the efects of the study interventions on LCI, again indicating that evaluation of the device in stable CF patients is required.

There were some diferences between the control and device groups at baseline that may have confounded our findings. The proportion of females was higher in the device group, which may explain the lower lung volumes in this group. Moreover, the degree of bronchial obstruction seemed to be more severe in the device group.

Despite these limitations, our study provides some of the first data on use of the new ACD in clinical practice. The device represents an interesting potential alternative therapy that appears to have activity in smaller airways and some benefits for patients with CF in terms of comfort and eficacy of the drainage session. These factors could be helpful in driving long-term adherence in this chronic disease population. Randomised trials of the device in larger patient populations followed over a longer period are planned.

In conclusion, the novel ACD is a promising new therapeutic option for patients with CF. It is safe, well tolerated, has good patient acceptability, and appears to improve drainage of the central and peripheral airways.
